# Native and engineered tropism of vectors derived from a rare species D adenovirus serotype 43

**DOI:** 10.18632/oncotarget.10800

**Published:** 2016-07-23

**Authors:** Natalya Belousova, Galina Mikheeva, Chiyi Xiong, Loren J. Stagg, Mihai Gagea, Patricia S. Fox, Roland L. Bassett, John E. Ladbury, Michael B. Braun, Thilo Stehle, Chun Li, Victor Krasnykh

**Affiliations:** ^1^ Department of Cancer Systems Imaging, The University of Texas MD Anderson Cancer Center, Houston, Texas, USA; ^2^ Department of Biochemistry and Molecular Biology, The University of Texas MD Anderson Cancer Center, Houston, Texas, USA; ^3^ Department of Veterinary Medicine and Surgery, The University of Texas MD Anderson Cancer Center, Houston, Texas, USA; ^4^ Department of Biostatistics, The University of Texas MD Anderson Cancer Center, Houston, Texas, USA; ^5^ Center for Biomolecular Structure and Function, The University of Texas MD Anderson Cancer Center, Houston, Texas, USA; ^6^ The University of Texas Graduate School of Biomedical Sciences at Houston, Houston, Texas, USA; ^7^ Interfaculty Institute of Biochemistry, University of Tuebingen, Tuebingen, Germany; ^8^ Current address: University of Leeds, Leeds, United Kingdom

**Keywords:** adenovirus, serotype, tropism, CD46, targeting

## Abstract

Unique molecular properties of species D adenoviruses (Ads)—the most diverse yet underexplored group of Ads—have been used to develop improved gene vectors. The low seroprevalence in humans of adenovirus serotype 43 (Ad43), an otherwise unstudied species D Ad, identified this rare serotype as an attractive new human gene therapy vector platform. Thus, in this study we wished to assess biological properties of Ad43 essential to its vectorization. We found that (1) Ad43 virions do not bind blood coagulation factor X and cause low random transduction upon vascular delivery; (2) they clear host tissues more quickly than do traditionally used Ad5 vectors; (3) Ad43 uses CD46 as primary receptor; (4) Ad43 can use integrins as alternative primary receptors. As the first step toward vectorization of Ad43, we demonstrated that the primary receptor specificity of the Ad43 fiber can be altered to achieve infection via Her2, an established oncotarget. Whereas this modification required use of the Ad5 fiber shaft, the presence of this domain in chimeric virions did not make them susceptible for neutralization by anti-Ad5 antibodies.

## INTRODUCTION

Most adenovirus(Ad)-based vectors for human gene therapy developed to date were derived from human Ad serotype 5 (Ad5)—the best studied of all types. However, Ad vector development benefitted significantly from studies of Ad serotypes other than Ad5. These studies identified the essential differences in molecular properties between various Ads and were instrumental in addressing major limitations of Ad5-based vectors, including these vectors' lack of target receptor specificity, inefficient transduction of many types of human cells, unfavorable biodistribution *in vivo*, and neutralization by anti-Ad5 antibodies (Abs) found in many humans. This research yielded important knowledge on the structure of the receptor-binding sites within Ads fiber proteins [1], the significance of fiber length [2] and flexibility for efficient transduction [3], differential interaction of various Ads' hexons with blood coagulation factors [4–6] and liver macrophages [7, 8], and great variations in levels of neutralizing Abs in humans against various Ad serotypes [9]. As a result, important practical milestones in vector development have been achieved, such as the design of Ad vectors ablated of undesirable native receptor specificities [1], reduced uptake by non-target tissues [5, 10, 11], reduced toxicity [11, 12], and altered immunogenicity profiles [13]. Despite these successes, Ad vectors better suited for gene therapy are yet to be developed, thus warranting the exploration of currently uncharacterized Ad serotypes.

Several improvements of Ad vectors resulted from studies of species D Ads [5, 6, 10, 13], the most diverse and largely underexplored Ad species. Although complete genomic sequences are available for more than 40 members of this species (summarized at http://www.vmri.hu/~harrach/AdVtaxlong.htm), few of these Ads have been studied in significant detail to support their use for therapeutic gene delivery. Primary receptors have been identified for only a few species D Ads [14–17]; no systematic studies have been performed on these Ads' intracellular trafficking, assembly, and maturation; and data on these Ads' biodistribution and tropism *in vivo* are similarly limited [6, 7]. Only a few of these Ads have been tested as vectors [14, 18–21], and no attempts to alter these vectors' natural tropism in order to target gene delivery have been reported.

Previous report on low seroprevalence in humans of Ad serotype 43 (Ad43) [9]—an otherwise unexplored member of species D—makes this virus a candidate as an alternative platform for the generation of vectors capable of evading neutralization by pre-existing anti-Ad5 Abs found in most humans [9]. Thus, in this study we wished to take a first look at the important aspects of Ad43 biology directly relevant to future vectorization of this yet virtually unknown virus. To this end, we sequenced and annotated the genome of Ad43, compared its structure with those of other Ads, ascertained the biodistribution of intravenously injected Ad43 virions, designed a plasmid-based system that facilitates molecular manipulations with Ad43 genome, identified Ad43's primary receptors, and successfully modified the primary receptor specificity of Ad43 fiber to enable infection via human epidermal growth factor receptor type 2 (Her2), a recognized oncotarget. The results of this work lay the foundation for future development of Ad43-based vectors suitable for human gene therapy.

## RESULTS

### Owing to the lack of blood coagulation factor X (FX) binding by the Ad43 hexon, intravenously injected Ad43 vector causes significantly reduced off-target transduction

The global pairwise alignment of the Ad43 genome (GenBank accession number KC529648) with genomes of other species D Ads revealed a high homology (93–98%), whereas its alignment with genomes of species A, B, C, E, and F Ads showed much lower homology (40% to 70%). The E3 region and genes of the major capsid proteins of Ad43—the penton base, hexon, and fiber—diverged the most from those of all other Ad serotypes except Ad28 (Supplementary Figure S1 and Table S1). Because these major capsid proteins play essential roles in Ads' infection [5, 22, 23], we studied the effects of this divergence on Ad43 tropism.

**Figure 1:**
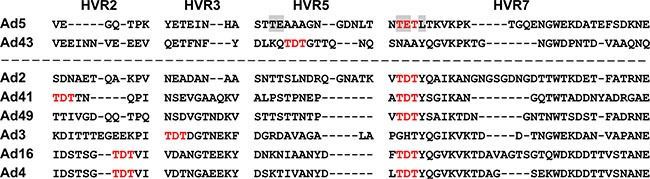
Alignment of Ad43 hexon HVRs 2, 3, 5, and 7 with HVRs of FX-binding hexons The Ad5 hexon amino acids residues in HVR5 and HVR7 involved in binding FX [10] are highlighted in gray; none of them is present in the Ad43 hexon. Shown in bold red are the TET tripeptide (in the Ad5 hexon's HVR7) previously implicated in FX binding [5] and its homologue, TDT, whose presence in HVRs of other Ads hexons correlates strongly with reported binding to FX. Of the human Ad serotypes whose binding to FX was tested and confirmed [5], seven contain TDT in their hexons. Six of these Ads are shown below the dashed line.

**Figure 2:**
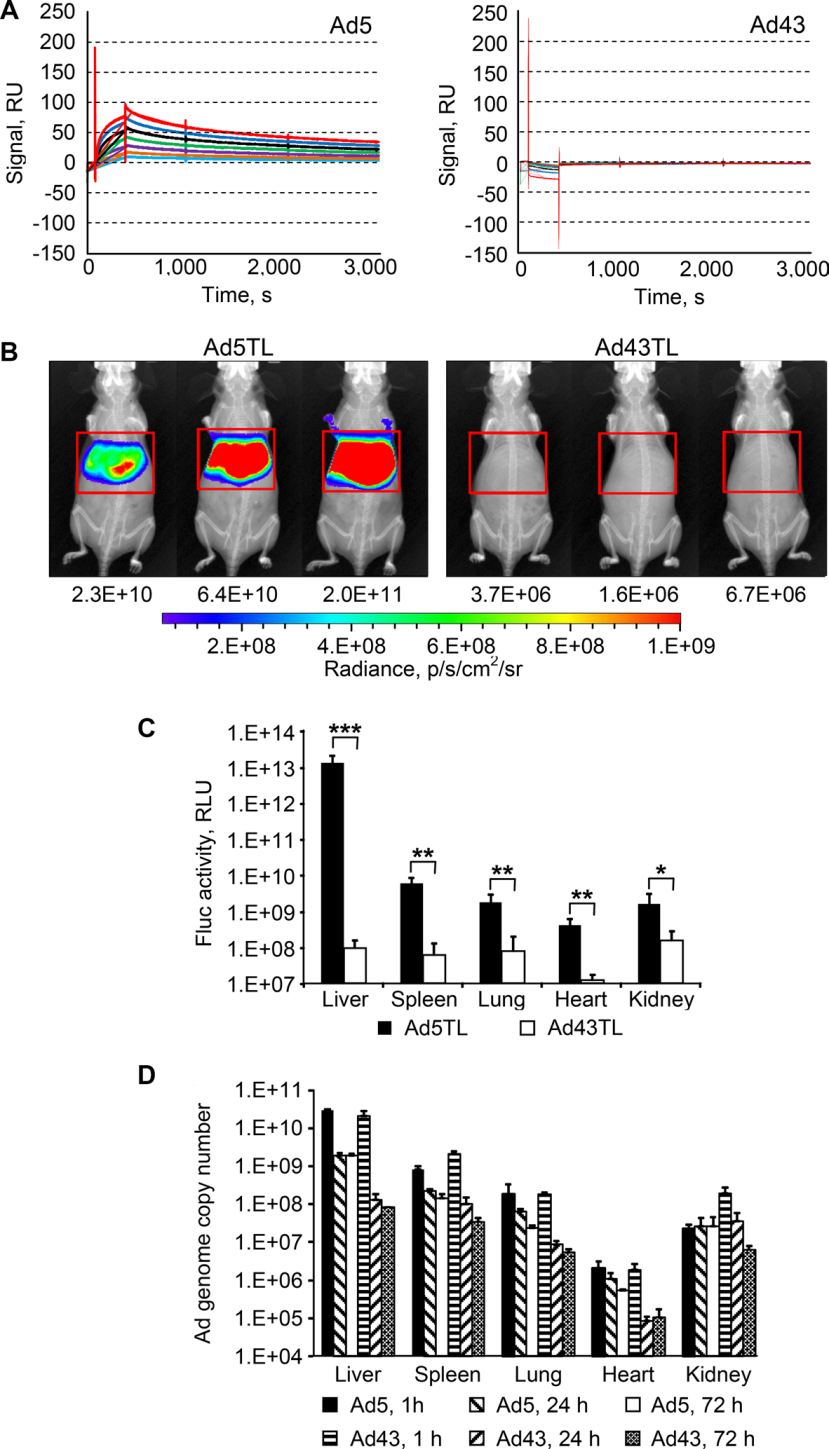
Lack of interaction between Ad43 virions and FX results in minimal hepatic transduction, but does not affect the vector uptake by the liver (**A**) Surface plasmon resonance analysis of FX interaction with Ad43 and Ad5. Sensorgrams obtained by probing chip-immobilized Ad virions with soluble FX are shown in duplicates, each curve corresponding to a separate run. Serial two-fold dilutions of FX covered a range of concentrations from 0.4 μg/ml (shown in light blue) to 25 μg/ml (shown in red). The calculated K_D_ for Ad5-FX interaction is 1.50 × 10^−9^ M. (**B**) Overlays of the whole-body bioluminescent and X-ray images of groups of mice (*n* = 3) intravenously injected with Ad5TL and Ad43TL vectors (4 × 10^10^ viral particles [VP] each) acquired 48 h after injections. The regions of interest that outline the livers are shown with red frames. The levels of bioluminescence measured in these regions of interest (total flux, photon/s) are shown below the images of mice. The pseudo-colour scale shows the radiance in photons/second/centimeter^2^/radian^2^. (**C**) The activity of the Fluc reporter measured in the homogenates of organs collected from Ad-injected mice 72 h after vectors injections. Each bar represents an average signal intensity in relative light units (RLU) per entire organ. Error bars indicate the standard deviations for a group of three animals; the measurements were taken in duplicates. The Fluc activities in PBS-injected mice were (in RLU/organ): 1.9 × 10^7^ (liver), 5 × 10^6^ (spleen), 9.9 × 10^5^ (lung), 1.2 × 10^6^ (heart), 3.4 × 10^6^ (kidney). *p < 0.05, **p < 0.01, ***p < 0.001. (**D**) The copy numbers of viral genomes shown here were measured using qPCR in organs isolated from mice 1 h, 24 h, or 72 h after injections with Ad5TL or Ad43TL (4 × 10^10^ VP). In panels (C and D) each bar represents an average copy number of viral genomes per organ and error bars show the standard deviations for each group animals (*n* = 3). *P* values for panel (D) are presented in the main text.

Our sequencing data revealed that the Ad43 hexon does not contain amino acids (aa) whose presence in the Ad5 hexon enables binding of FX, leading to undesired, off-target liver transduction by Ad5 vectors on intravascular delivery [6, 10] (Figure 1). Interestingly, however, the Ad43 hexon's hypervariable region (HVR) 5 contains a TDT-tripeptide whose presence in HVRs 2, 3, and 7 in other Ad hexons strongly correlates with FX binding [5, 6] (Figure 1). Our assessment of Ad43 interaction with FX by surface plasmon resonance showed no measurable association whereas an interaction between Ad5 and FX is apparent (Figure 2A).

This lack of association between FX and Ad43 predicted negligible hepatic transduction by an intravenously administered Ad43. Indeed, the patterns of liver transduction in mice injected with Ad43TL vector and in mice injected with Ad5TL vector—the E1-deleted Ads each expressing a genetic fusion of the herpes simplex virus thymidine kinase and firefly luciferase (TL)—differed dramatically: on average, transgene reporter bioluminescence activity in Ad43TL-injected mice was at background level and 2.4 × 10^4^ times lower than such activity in Ad5TL-injected animals (Figure 2B). The measurements of luciferase activity in the lysates of livers of Ad-injected mice collected 24 h later revealed a 1.3 × 10^5^-fold difference (Figure 2C). Notably, transduction of all other tested organs was significantly lower in mice injected with Ad43TL than in Ad5TL-injected animals (Figure 2C and Table 1).

**Table 1:** Reporter activity in tissues isolated from Ad-injected mice

Vector	Liver		Spleen		Lung		Heart		Kidney	
	Per organ^a^	Per gram^b^	Per organ	Per gram	Per organ	Per gram	Per organ	Per gram	Per organ	Per gram
Ad5TL	1.3 × 10^13^	9.9 × 10^12^	6.0 × 10^9^	5.8 × 10^10^	1.8 × 10^9^	7.4 × 10^9^	4.1 × 10^8^	2.9 × 10^9^	1.6 × 10^9^	5.4 × 10^9^
Ad43TL	9.6 × 10^7^	7.2 × 10^7^	6.2 × 10^7^	6.0 × 10^8^	7.9 × 10^7^	3.2 × 10^8^	1.2 × 10^7^	8.1 × 10^7^	1.5 × 10^8^	4.9 × 10^8^
Signal ratio^c^	1.3 × 10^5^	1.4 × 10^5^	9.6 × 10	9.7 × 10	2.2 × 10	2.3 × 10	3.3 × 10	3.6 × 10	1.0 × 10	1.1 × 10
PBS^d^	1.9 × 10^7^	2 × 10^7^	1.5 × 10^6^	1 × 10^7^	9.9 × 10^5^	9.6 × 10^6^	1.2 × 10^6^	9.6 × 10^6^	3.4 × 10^6^	9.6 × 10^6^

a,b Activity of the reporter averaged for groups of three animals is shown in RLUs per entire organ and per gram of tissue.

c Ratio of bioluminescence signals measured in Ad5TL-injected mice to those measured in Ad43TL-injected mice.

d Luciferase activity measured in tissues isolated from phosphate-buffered saline-injected mice.

### Ad43 virions quickly clear non-target tissues

*In vivo* distribution patterns and temporal dynamics of these patterns are essential parameters of vector clearance by the host. To determine them for Ad43, we measured the copy numbers of viral genomes in tissues collected from mice 1, 24, and 72 h after tail vein injections with Ad43TL and control Ad5TL. One hour after injection (Figure 2D), the profiles of biodistribution for both Ads were similar. Differences were seen only in the spleen (*p* = 0.0056) and in the kidneys (*p* = 0.0012), in which Ad43 was found at level 2.6 times and 8.5 times higher than that of Ad5, respectively. Despite the drastic difference seen in the liver transduction patterns, the levels of both Ads in the liver at this time point were very similar and much higher than in any other tissue (*p*-values for Ad43 and Ad5 are < 0.0004 and < 0.0003, respectively).

Notably, over the next 24 h and 72 h, the Ad43 genome copy numbers decreased faster than did those of Ad5 genome (*p* < 0.0001). In particular, over the course of the 24 h period after injection, the amount of Ad DNA in the liver had decreased dramatically for both Ads and remained unchanged for each vector 48 h later. Importantly, however, the magnitude of this change was substantially different for the two compared Ads. The Ad5 DNA content in the liver decreased 15 times at the 24 h time point but remained higher than in any other tissue. The level of Ad43 DNA in the liver decreased 163 times and became indistinguishable from that in the spleen (*p* = 0.4861), and only slightly higher than that in the kidneys (*p* = 0.0415), in which Ad43 DNA also decreased substantially relative to the 1 h time point values. The amount of Ad43 DNA decreased significantly between the 1 h and 24 h time points for all tissues: the liver, spleen, lungs, heart, and kidneys (all *p*-values < 0.02). These decreases were significantly larger for Ad43 than for Ad5 in all tested tissues (all *p*-values < 0.02).

Comparison of the data for the 24 h and 72 h time points revealed further decreases in Ad43 DNA content in the spleen (*p* = 0.0480) and kidneys (*p* = 0.0282), and in Ad5 DNA content in the spleen (*p* = 0.0485), heart (*p* = 0.0416), and lungs (*p* = 0.0021). Although the Ad43 genome copy number in the kidney gradually decreased between 1 h and 72 h, the Ad5 DNA copy number stayed relatively constant over time.

### Ad43 exploits two types of receptors for infection

**Figure 3:**
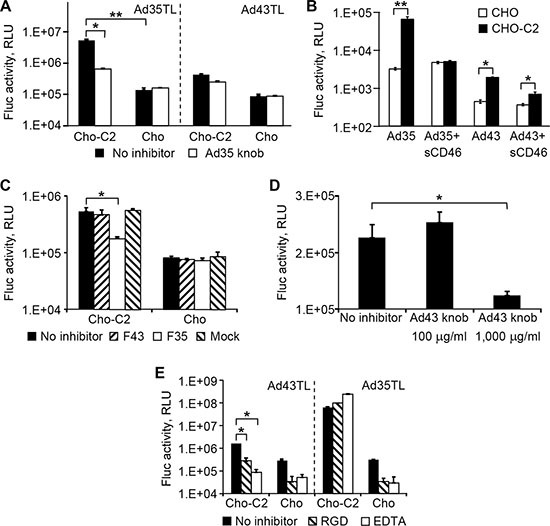
Ad43 uses CD46 and integrins as primary receptors (**A**) The CHO-C2 and CHO cells (CD46-positive and CD46-negative, respectively) were infected with Ad43TL and Ad35TL vectors. CD46-specific inhibition of transduction by both viruses was achieved by blocking the receptor with a recombinant Ad35 knob prior to addition of virus. Here and in panels **B**–**E** the activity of the Fluc reporter in cell lysates is shown in RLUs as the mean of three replicates; error bars show the standard deviations. (**B**) Prior to infection of CHO-C2 and CHO cells, Ad43TL and Ad35TL were preincubated with sCD46 used here as a competitor of cell-associated CD46. (**C**) The inhibition of Ad43TL transduction with the Ad43 and Ad35 fibers. Prior to infection with Ad43TL, CHO-C2 and CHO cells were pre-incubated with the lysates of 293F43, 293F5, or 293 cells expressing the Ad43 fiber (F43), Ad35 fiber (F35), or no fiber (mock), respectively. “No inhibitor” bars show the transduction levels seen in the absence of cell lysates. The amount of lysates was normalized on the basis of fiber concentrations measured by western blotting. The control cells were infected with the virus in the plain media (no inhibitor). Fluc activity was measured in cell lysates 24 h after infection. (**D**) The inhibition of CHO-C2 cell transduction by the Ad43 vector with the recombinant Ad43 knob. Prior to being infected with Ad43TL, CHO-C2 cells were incubated with the recombinant Ad43 knob at concentrations of 100 μg/ml and 1,000 μg/ml. Fluc activity was measured in cell lysates 24 h after infection. (**E**) The CHO-C2 and CHO cells were pre-incubated with the integrin-binding GRGDSP-peptide (shown as “RGD”) or EDTA on ice to block binding with integrins and then incubated with Ad43TL or Ad35TL (control) on ice to prevent internalization of the virus. The unbound Ad particles were removed by washing with ice-cold medium, and the cultures were transferred to 37°C to allow for internalization of cell-bound virions. No pretreatment is shown as “no inhibitor”. After unbound virions were washed away, the cells were incubated at 37°C to allow virus entry. Fluc activity was measured in cell lysates 24 h after infection. *p < 0.05, **p < 0.01.

Most Ads attach to their primary receptors on cells using the knob domain of the fiber protein. Several receptors for species D Ads, such as CD46 [14, 19, 20], sialic acid [24, 25], and coxsackievirus and Ad receptor (CAR) [26, 27], are known. To identify the primary receptor(s) for Ad43, we first tested whether Ad43 uses CD46 for infection. To this end, CD46-negative CHO cells and CHO-C2 cells that express the C2 isoform of CD46 were infected with Ad43TL and Ad35TL (used here as positive control because of Ad35's known specificity for CD46 [28]). Prior to infection, the cells were incubated with the recombinant Ad35 fiber knob domain—a strong competitive blocker of CD46. As anticipated, Ad35TL transduced CHO-C2 cells much more efficiently than it transduced CHO cells (49-fold), and the knob inhibited Ad35 transduction of CD46-positive cells very effectively, whereas no inhibition was seen in control CHO cells (Figure 3A). A similar dependence on CD46 was also seen for Ad43TL: CHO-C2 cells were transduced by Ad43TL 5.3-fold more efficiently than were CHO cells, thus supporting the use of CD46 by Ad43. Notably, although Ad43TL transduced CHO-C2 cells less efficiently than did Ad35TL, inhibition of this transduction by the Ad35 fiber knob was also lower (45% *vs* 90%).

Dependence of Ad43 infection on CD46 was further demonstrated in a similar experiment in which soluble CD46 protein (sCD46) was tested as infection inhibitor. In this experiment the viruses (Ad35TL or Ad43TL) were preincubated with sCD46 prior to be added to cells (CHO or CHO-C2). Under these conditions, sCD46 blocked 92% of CHO-C2 transduction by Ad35TL and 66% of transduction by Ad43TL; no inhibition of infection by sCD46 was seen in CHO cells (Figure 3B).

An additional proof of CD46 involvement in Ad43 infection was obtained by infecting CHO and CHO-C2 cells with Ad43 in the presence of full-sized Ad35 and Ad43 fibers. Although none of the fibers blocked transduction of CHO cells by either virus (Figure 3C), transduction of CHO-C2 cells by Ad43 was inhibited by the Ad35 fiber but not by the Ad43 fiber. These data confirmed our initial findings of the CD46-dependence of Ad43 infection and also showed that, at the tested concentrations, the free Ad43 fiber is unable to compete for CD46 attachment with the virion-associated fibers. The inefficient inhibition of Ad43 infection of CHO-C2 cells with the recombinant Ad43 fiber knob protein provided additional evidence of the fiber's inability to compete: the knob did not inhibit infection of CHO-C2 cells at 100 mg/ml and caused only moderate inhibition (55%) at the very high concentration of 1 mg/ml (Figure 3D).

The Ad35 fiber's knob is known to have very high affinity for CD46 [29] and is thus a strong competitor. The incomplete inhibition of Ad43 infection by the CD46-binding Ad35 fiber and its knob suggested that molecules other than CD46 may function as alternative receptors for Ad43. This possibility was supported by previous reports on the use by some Ads of more than one type of molecule as a primary receptor. For example, CD46, CD80, CD86, and desmoglein 2 were identified as receptors for Ad3 [30–32].

We also knew that Ad9, a member of species D, uses its penton base protein to attach directly to integrins and achieve successful infection, thereby obviating the need for fiber-receptor interaction [16, 33]. To test whether a similar cell attachment mechanism is used by Ad43, virus binding to integrins on CHO and CHO-C2 cells was blocked by pretreating the cells with an RGD-containing peptide or EDTA prior to infection with Ad43TL. Under these conditions, both inhibitors blocked more than 80% of Ad43 transduction of each cell line, suggesting that, irrespective of CD46 expression, the virus can use integrins for attachment and infection (Figure 3E).

### Modification of Ad43's primary receptor specificity using an Ad43/5 fiber chimera yields a Her2-specific vector unaffected by Ad5-neutralizing antibody

**Figure 4:**
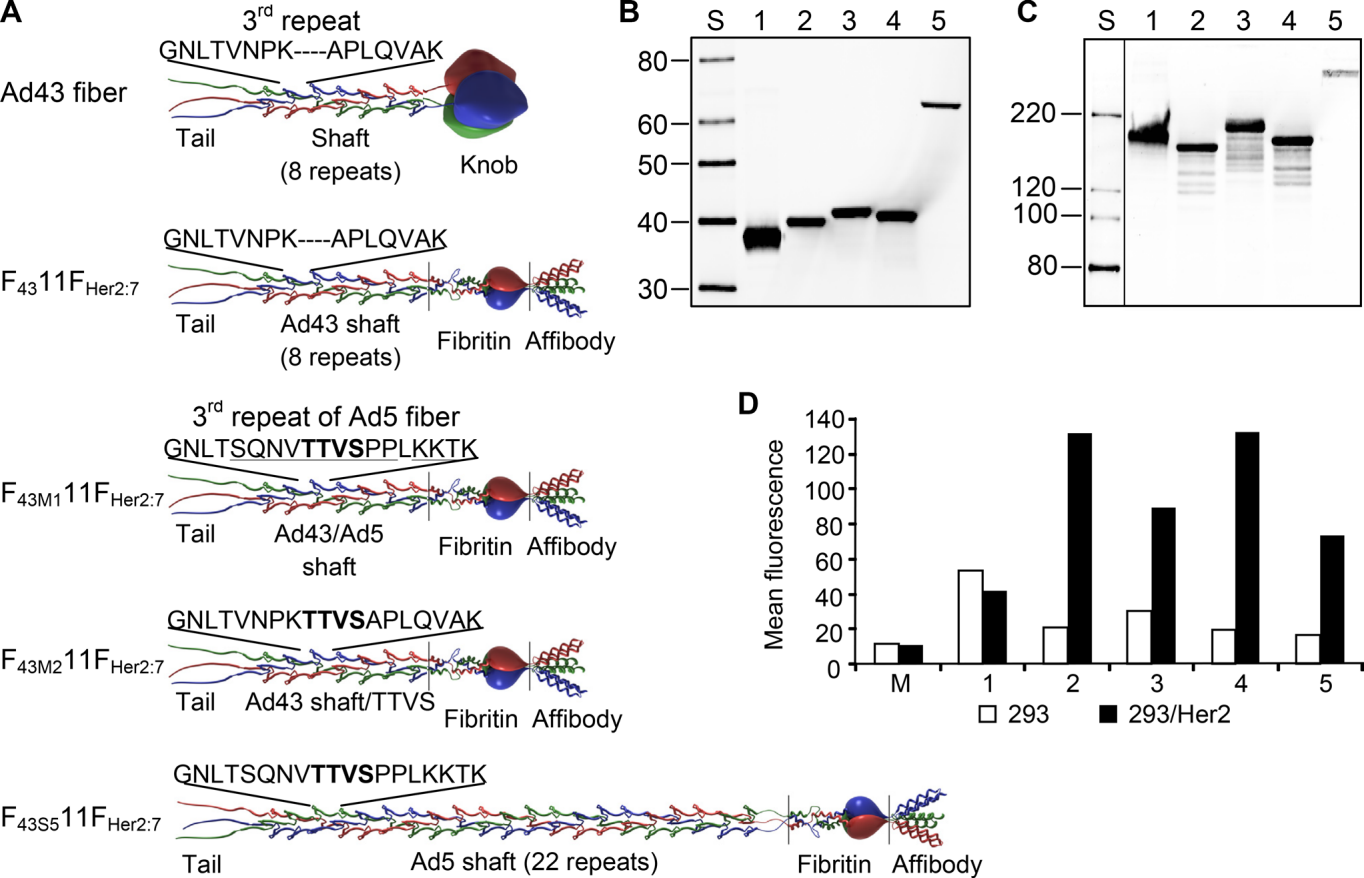
Ad43 fiber-derived Her2-targeting chimeras form trimers and bind to Her2 (**A**) The structure of the Ad43 fiber and the Ad43 fiber-derived chimeras. The Ad43 fiber consists of the tail, shaft, and knob domains. The Ad43 fiber shaft is short (8 repeats) and it lacks a TTVS hinge motif, whose presence in the third pseudo-repeat of the long Ad5 fiber shaft (22 repeats) make the Ad5 fiber flexible. In all chimeras, the tail domain was left intact, whereas the knob domain was replaced with the trimerization domain of phage T4 fibritin protein and a Her2-targeting affibody, Zher2:7. The F_43_11F_Her2:7_ chimera contains an unmodified Ad43 fiber shaft. To make the chimeras flexible, in F_43M1_11F_Her2:7_, the entire third repeat of the Ad43 fiber shaft was replaced with the third repeat of the Ad5 fiber shaft and in F_43M2_11F_Her2:7_, the TTVS hinge was inserted into the Ad43 fiber's third repeat. To make the chimera flexible and to simultaneously elongate it, the Ad43 fiber shaft in F_43S5_11F_Her2:7_ was replaced with the Ad5 fiber shaft. The TTVS hinge is shown in bold; the amino acids in the Ad5 shaft third repeat that differ from those in Ad43 are underlined. Western blot of boiled (panel **B**) and non-boiled (panel **C**) lysates of 293T cells transiently expressing the fiber chimeras: F_43_11F_Her2:7_ (lane 2), F_43M1_11F_Her2:7_ (lane 3), F_43M2_11F_Her2:7_ (lane 4), and F_43S5_11F_Her2:7_ (lane 5); the wt Ad43 fiber is in lane 1. (Here and in other panels showing the results of western blotting and SDS-PAGE, lane S shows protein standards with the molecular masses in kDa). All the chimeras formed trimers but were expressed at lower levels than was the wt Ad43 fiber. The predicted molecular masses of monomeric wt Ad43 fiber, F_43_11F_Her2:7_, F_43M1_11F_Her2:7_, F_43M2_11F_Her2:7_, and F_43S5_11F_Her2:7_ are 39.4, 36.8, 37.3, 37.2, 59.4 kDa, respectively. The image shown in panel 4C has been cropped and regrouped; the lane showing protein standards was digitally enhanced. (**D**) Flow cytometry of Her2-positive and negative cells probed with the designed fiber chimeras. The protein probes are: wt Ad43 fiber (control), 1; F_43_11F_Her2:7_, 2; F_43M1_11F_Her2:7_, 3; F_43M2_11F_Her2:7_, 4; F_43S5_11F_Her2:7_, 5; lysate of mock transfected 293T cells, M.

The fact that Ad43 uses at least two types of broadly expressed receptors for infection makes targeted therapy with Ad43-based vectors unlikely unless the virus can be made target-specific. As a first step in this direction, we wished to test the feasibility of such tropism modification by directing Ad43 to human epidermal growth factor receptor type 2 (Her2), a known tumor biomarker, by modifying the virus' fiber protein. The previous reports on the importance of Ad fiber length and flexibility to efficient transduction [2, 3] and the shortness and predicted rigidity of the Ad43 fiber revealed by our sequencing data collectively suggested that successfully targeting this fiber may require elongation and engineering of additional flexibility. To these ends, we designed four Ad43 fiber-derived, Her2-targeted chimeras each containing the amino-terminal tail domain of the Ad43 fiber (aa 1–48), the carboxy-terminal domain of the fragment of phage T4 fibritin protein that comprises the last two α-helical repeats of the stalk and the foldon domain (aa 393–487, GenBank accession number L43611), the (Gly_4_Ser)_3_ linker, and the Her2-specific affibody Z_her2:7_ [34] (Figure 4A). The central domain contained a non-modified Ad43 shaft (Ad43 fiber aa 49–180) in a F_43_11F_Her2:7_ chimera, an Ad43 shaft with its 3rd repeat (Ad43 fiber aa 76–90) replaced with the 3rd repeat of the Ad5 fiber shaft (Ad5 fiber aa 74–94) in F_43M1_11F_Her2:7_, an Ad43 shaft with insertion of a TTVS-tetrapeptide between V83 and P84 of F_43M2_11F_Her2:7_, and an Ad5 shaft (Ad5 fiber aa 50–398) in F_43S5_11F_Her2:_7.

**Figure 5:**
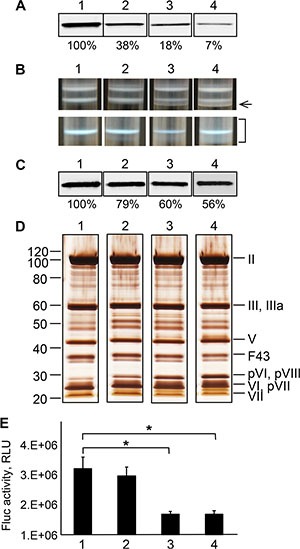
Characterization of the Ad43TL virions amplified in cell lines stably expressing the Ad43 fiber at various levels (**A**) Western blot of the lysates of 293F43 clones expressing the wt Ad43 fiber. Lane 1, clone 20 with a maximal level of fiber expression (set at 100%); lanes 2, 3 and 4, clones 7, 3, and 2, respectively, each expressing the fiber at 38%, 18% and 7% of expression in clone 20, respectively. (**B**) The appearance of the viral bands after the first (upper row) and second (lower row) centrifugation in CsCl. The tubes 1, 2, 3, and 4 contain samples of Ad43TLΔF amplified in 293F43 clones 20, 7, 3, and 2, respectively. Only the sample obtained from clone 20 produces the expected banding patterns. The arrow on the right points at additional, higher-density bands in viral preparations obtained from clonal lines 7, 3, and 2. The bracket shows an area of the gradient containing a diffuse area corresponding to heterogeneous viral particles isolated from line 2. Western blot (panel **C**) and silver staining (panel **D**) of purified virions (10^10^ VP) shown in panel B. The Ad43TLΔF virions propagated in clones with lower levels of fiber expression contain fewer fibers (as seen in panel C) and more unprocessed precursors of the proteins pVI, pVII, and pVIII (panel D). Lanes 1-4, viruses collected from tubes 1–4 (as shown in panel B), respectively. The Ad43 structural proteins and their predicted migration positions are shown on the right of panel D. Here and in Figures 6 and 7, F43 indicates wt Ad43 fiber. Panels A–D show cropped images. (**E**) Gene transfer by purified viruses. Shown are the activities of Fluc in lysates of 293 cells infected with viruses at an MOI of 100 VP/cell (*n* = 3), which were measured 24 h after infection. Error bars indicate the standard deviations. *p < 0.05.

The chimeras and control wild-type (wt) Ad43 fiber were transiently expressed in 293T cells to compare their expression levels and test their binding to Her2 and self-trimerization—the property essential for fiber incorporation in virions. Western blotting of these proteins showed significant variations in their expression (Figure 4B); it also showed that all non-denatured chimeras formed trimers (Figure 4C). Flow cytometry in which the Her2-positive 293/Her2 and Her2-negative 293 cells were probed with these chimeras showed much more efficient binding of these proteins to 293/Her2 cells than to Her2-negative 293 cells (Figure 4D). However, the observed significant decrease in chimeras expression was a concern, because it could result in inefficient incorporation of these chimeras in Ad43 virions and decreased infectivity. Also, low expression of the fiber could lead to incomplete maturation of viral proteins pVI, pVII, and pVIII, thereby yielding noninfectious virions, as has been previously demonstrated for fiberless Ad5 [35]. To assess these risks, we first studied the dependence of Ad43 fiber incorporation, and virions' maturation, and infectivity on fiber expression efficiency. To this end, we infected the sublines of the 293-derived cell line 293F43 each expressing wt Ad43 fiber at a different level (Figure 5A), purified the viral progeny (Figure 5B) and characterized it by using western blotting (Figure 5C), protein electrophoresis (Figure 5D), and transduction of 293 cells (Figure 5E). Importantly, this study showed strong correlation between overall fiber expression in Ad43-infected cells, on the one hand, and both the efficacy of fiber incorporation and completeness of virions' maturation (Figure 5A–5D), on the other hand. It also showed, however, that even at low levels of fiber production, virus infectivity decreases insignificantly (Figure 5E). Together, these findings suggested that even at reduced expression levels, the fiber chimeras might be able to support Her2-mediated infection by the virus.

**Figure 6:**
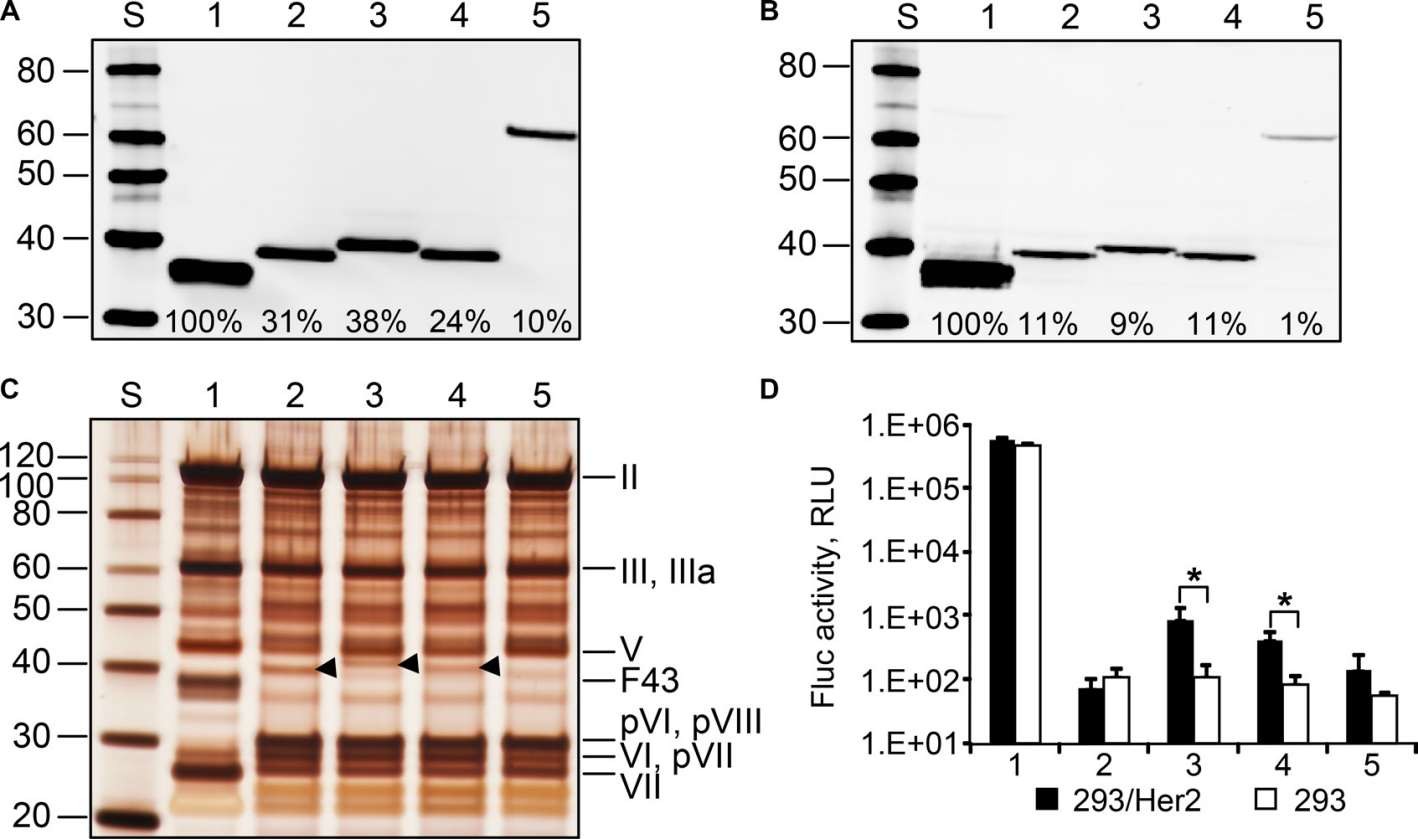
Ad43 vectors encoding chimeric fibers incorporate them at reduced efficiency, contain unprocessed precursor proteins, and poorly transduce Her2-expressing cells In all panels: 1, Ad43TL; 2, Ad43TL.F_43_11F_Her2:7_; 3, Ad43TL.F_43M1_11F_Her2:7_; 4, Ad43TL.F_43M2_11F_Her2:7_; 5, Ad43TL.F_43S5_11F_Her2:7_. Western blot of purified viruses (panel **A**) and lysates of 293A cells infected with the fiber-modified viral vectors (panel **B**). Shown are percentages of the chimeras' incorporation and expression relative to those of wt Ad43 fiber (set at 100%). (**C**) SDS-PAGE gel (silver stained) of purified viruses. Arrowheads indicate the positions of chimeras F_43_11F_Her2:7_, F_43M1_11F_Her2:7_, and F_43M2_11F_Her2:7_. (**D**) The gene transfer to 293/Her2 and 293 cells with purified viruses. The cells were infected with purified viruses at an MOI of 100 VP/cell. Mean values for Fluc activity (*n* = 3) in cells 24 h after infection are shown. Error bars indicate the standard deviations. *p < 0.05.

Once Ad43TL-derived vectors encoding Her2-targeting chimeras had been prepared, our western blotting of these Ads showed that all chimeras incorporated in viral particles less efficiently than did the wt Ad43 fiber (Figure 6A), correlating with overall chimera expression levels, which were reduced 10- to 100-fold (Figure 6B). Analysis of the viruses' protein composition revealed that all contained significant amounts of unprocessed pVI, pVII, and pVIII precursor proteins (Figure 6C). These results were in agreement with our previous data on the effects of reduced wt Ad43 fiber expression on virus protein composition; however, in contrast to the viruses containing decreased numbers of wt Ad43 fibers—which transduced cells quite efficiently (Figure 5E)—the chimera-containing viruses showed low levels of overall gene transfer, with the Her2-dependent component negligible (Figure 6D). This low level of Her2-dependent transduction by Ad43TL.F_43S5_11F_Her2:7_ was completely unexpected because Ad43TL.F_43S5_11F_Her2:7_ encoded the chimera that was structurally very similar to the Ad5 fiber-based chimera 11F_Her2:7_, which had been designed previously and proved fully functional [36]. It should be noted, however, that in contrast to F_43S5_11F_Her2:7_, the 11F_Her2:7_ chimera incorporated in Ad5 virions very efficiently. The observed 10-fold decrease (compared with wt fiber) in F_43S5_11F_Her2:7_ incorporation (Figure 6A) meant that, on average, an Ad43TL.F_43S5_11F_Her2:7_ particle contained only 1.2 copies of the chimera. Calculations using a binomial distribution model predicted that 28% of virions in the preparation contained no fibers, 38% and 23% contained one or two fibers, respectively, and less than 0.06% contained six fibers or more.

**Figure 7:**
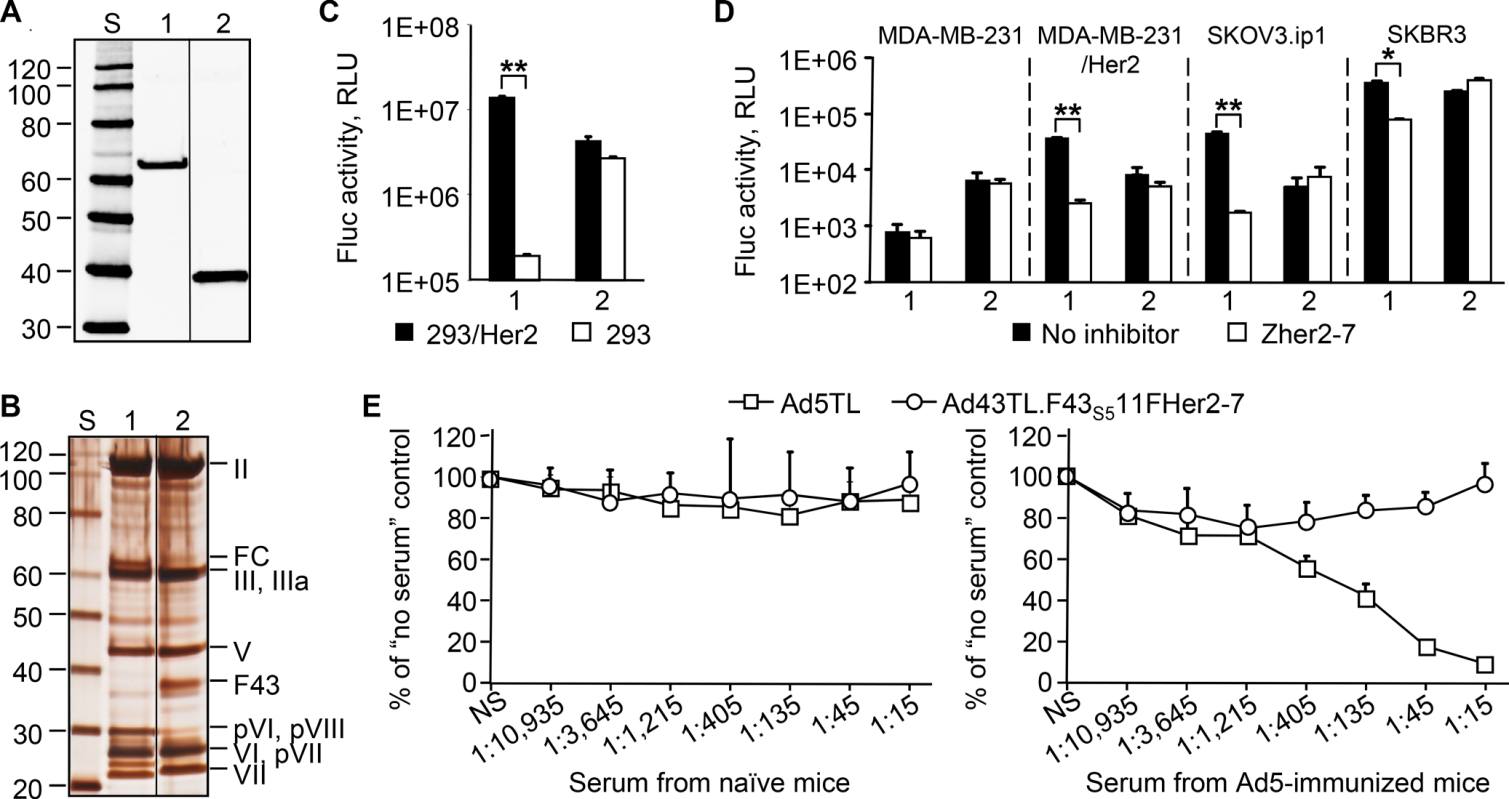
Increased expression of the F_43S5_11F_Her2:7_ chimera yields an Ad43 vector that is capable of efficient Her2-specific transduction and is not neutralized by anti-Ad5 Abs The western blotting (panel **A**) and SDS-PAGE (panel **B**) of CsCl-purified virions (10^10^ VP/lane) of Ad43TL.F_43S5_11F_Her2:7_ amplified in 293F_43S5_11F_Her2:7_ cells (lane 1), and Ad43TL (lane 2). FC, the F_43S5_11F_Her2:7_ fiber chimera. The images shown in panel 7A and 7B have been cropped and regrouped. The gene transfer to 293/Her2 and 293 cells (panel **C**), and tumor cell lines expressing Her2 (panel **D**). The Zher2-7 affibody was used as a Her2-specific inhibitor (panel D only). Ad43TL.F_43S5_11F_Her2:7_, 1; Ad43TL, 2. The Her2-negative MDA-MB-231 cells, a parental line for Her2-expressing MDA-MB-231/Her2 cells, are shown as a control. (**E**) *In vitro* vector neutralization assay. The sera obtained from naïve or Ad5-immunized mice were added to 293/Her2 cells prior to transduction with Ad5TL or Ad43TL.F_43S5_11F_Her2:7_ vectors. The levels of transgene expression in transduced cells relative to expression level seen in the absence of serum are shown. The sera dilution factors are shown below the graphs. NS, no serum. In panels C, D, and E, data are shown as the mean of three replicates; error bars show the standard deviations. *p < 0.05, **p < 0.01.

To see whether efficient Her2-dependent transduction by an Ad43 vector could be achieved by increasing the expression of F_43S5_11F_Her2:7_, we generated a 293F_43S5_11F_Her2:7_ cell line stably expressing this chimera at a high level. The Ad43TL.F_43S5_11F_Her2:7_ vector was remade by infecting these cells with Ad43TL.ΔF previously amplified in wt Ad43 fiber-expressing 293F43 line 20 cells. The increased expression of the chimera in 293F_43S5_11F_Her2:7_ cells yielded Ad43 particles that contained F_43S5_11F_Her2:7_ at the level of 49% of wt Ad43 fiber in control Ad43TL particles (Figure 7A). Also, compared to the Ad43TL.F_43S5_11F_Her2:7_ virions previously obtained from 293A cells (Figure 6C), this new preparation of vector contained much lower amounts of unprocessed pVI, pVII, and pVIII precursor proteins (Figure 7B). As a result of these improvements, the virus gained an ability to transduce cells efficiently and in a Her2-dependent manner; gene transfer to 293/Her2 cells was 72-times more efficient than transfer to 293 cells, and 3-times more efficient than by unmodified control Ad43TL (Figure 7C). The advantage of Ad43TL.F_43S5_11F_Her2:7_ over Ad43 vector with native tropism was also shown using Her2-expressing tumor cell lines MDA-MB-231/Her2, SKOV3.ip1, and SKBR3 Figure 7D). In this instance, the Her2-dependence of gene transfer was confirmed by selective inhibition of transduction with a free Her2-binding affibody competitor.

Importantly, the presence of a rather large Ad5-derived protein domain—the Ad5 fiber shaft—in Ad43TL. F_43S5_11F_Her2:7_ particles has not made this Ad43 vector susceptible to neutralization by anti-Ad5 Abs (Figure 7E), suggesting that the Ad43 antigenic profile remains largely unchanged despite the presence of the Ad5 fiber shaft.

## DISCUSSION

Extensive epidemiological studies identified the preexisting humoral immunity to Ad5 found in many humans as one of the major limitations of Ad5-based gene therapy [9]. In addition, the strong immunogenicity of Ads and the need to administer vectors repeatedly to achieve clinically relevant outcomes strongly suggest that a successful gene therapy strategy based on a single Ad serotype is unlikely. This is because even in the absence of preexisting anti-Ad Ab, in response to vector injection the anti-vector humoral immunity builds quickly thus making successive rounds of therapy inefficient [37]. Therefore, the need for vectors with different antigenic profiles is apparent. The natural serodiversity of Ads and significant differences in Ads' seroprevalence in humans provide a rationale for designing vectors with altered antigenicity [13]. Importantly, the molecular diversity of Ad serotypes, which results in differential modes of Ad vector interaction with host tissues, may be used to solve the problem of off-target gene delivery by Ads and its resulting toxicity. For instance, previous studies have shown that the undesired hepatic transduction and toxicity of Ad5-based vectors can be significantly reduced by developing inter-serotype hexon chimeras [10, 11].

In this regard, our study of Ad43 interaction with FX and patterns of gene transfer on Ad43 vascular delivery provide additional evidence of an emerging concept that links weak or nonexistent FX binding by a given Ad with very low hepatic transduction by that virus. In agreement with recent reports [4–6], we have shown that Ad43's inability to cause liver transduction correlates well with its failure to associate with FX. Interestingly, comparison of Ad43's hexon structure with those of other Ads could not reliably predict the strength of Ad43-FX interaction; although the absence of key amino acids known to mediate such binding was predictive of a lack of binding, the presence of a putative FX binding-enabling TDT motif suggested otherwise. Our data clearly show that the mere presence of TDT motif in the hexon is not sufficient to enable FX binding. Supporting further development of Ad43-derived therapeutic vectors, the discovery of TLR/NF-kB-mediated innate immunity activation following cellular uptake of Ad-bound FX [12] suggests that the magnitude of this immune response to FX-nonbinding Ad43 and associated toxicity should be lower than responses triggered by Ad5 vectors.

Whereas the level of random transduction by systemic Ad43 was very low, we found the level of virus sequestration by the liver to be high. This is most likely owing to virus uptake by Kupffer cells—a mechanism well-established for every Ad serotype tested to date [4, 7]. Therefore, future development of Ad43 as a truly targeted vector will require ablation of its recognition by Kupffer cells—a universal Ad-vector development problem for which a satisfactory solution applicable to humans is yet to be found.

Of importance to potential clinical use of Ad43-based gene vectors is the observed accelerated clearance of this virus from the host's tissues. Although the molecular mechanisms underlying this clearance are yet to be identified, it is reasonable to speculate that in certain clinical scenarios this clearance may offer the advantage of a more time-limited genetic intervention than is possible with Ad5-based vectors.

We found that Ad43 can use two alternative receptors—CD46 and integrins—for infection. Together with the previous reports on Ad3, Ad5, Ad9, and Ad49 [14, 16, 30–32, 38, 39], this finding establishes the use of alternative cell entry pathways by an Ad serotype as an important evolutionary accomplishment that results in virus' enhanced infectivity, propagation, and survival.

The drastic difference in efficacy with which the Ad35 and Ad43 fiber knobs inhibited Ad43 infection that we observed is likely due to the differing affinities these knobs have for CD46. Supporting this explanation is the fact that, of all the Ad fiber knobs tested, the Ad35 knob is one of the tightest binders of CD46 (equilibrium dissociation constant, K_D_, of 16 nM [29]; K_D_s in the nanomolar range have been reported for other CD46-binding Ad fiber knobs [29, 40]). The poor inhibition of Ad43 transduction by the Ad43 fiber and its knob also suggests that their affinity for CD46 is too low for successful competition with the virus-associated fibers in which receptor-binding efficacy is dramatically enhanced by avidity. The gains of such cooperative binding to an Ad receptor have previously been shown for Ad3 [32], whose knob binds to desmoglein 2 with a very low affinity (K_D_ of 10 mM) [41]. Also, these gains are realized by the low-affinity CD46-binders Ad21 and Ad16 (K_D_s of 284 nM and 437 nM, respectively [40, 42]).

The high-avidity binding of Ads to primary receptors via low-affinity fibers has important practical implications for the development of targeted Ad vectors because it indicates that the affinity of a targeting ligand for a desired receptor does not have to be very high to support efficient infection. The feasibility of such relatively low affinity targeting of Ad5 has been demonstrated using the weakly binding Ad3 knob [43] and by modifying the tropism of Ad5 and simian Ad24 (sAd24), with a low-affinity affibody ligand [4, 36].

Importantly, our identification of CD46 as the primary receptor for Ad43 does not call into question the validity of our biodistribution studies done in mice, which don't express CD46 ubiquitously. This is because several *in vivo* studies of unmodified Ad virions in mice have convincingly shown that the observed virus distribution patterns are determined by the interactions of Ad virions with FX [5, 6, 10, 44], scavenger receptors on Kupffer cells, natural Abs, and complement [45] in which the Ad fiber's receptor specificity plays no known role. Given these findings, studying the biodistribution of unmodified Ad43 virions in CD46-expressing transgenic mice [46–48] does not seem to offer real benefits.

Applying the fiber knob replacement targeting approach [49] to Ad43 in this study revealed some of the limitations of this strategy: in contrast to Ad5 and sAd24 previously targeted using this strategy [4, 36], Ad43 could not be targeted to Her2 by simple replacement of its knob domain with the fibritin-affibody fusion. The predicted rigidity of the Ad43 fiber is an unlikely cause of this problem, because insertion of a flexible hinge did not rectify the chimera's deficiency. The desired tropism modification of Ad43 was successful only after replacement of the rigid and short Ad43 fiber shaft with a flexible and long Ad5 fiber shaft, suggesting that the length of the chimera (rather than its flexibility) is essential for Her2-dependent infection. Given the relatively low affinity of the Zher2-7 affibody for Her2 (K_D_=140 nM [34]), binding of Ad43 to Her2 may require simultaneous engagement of several ligands by a single Ad virion. In this scenario, the density of Her2 molecules on the cell surface may favor receptor cross-linking and infection only by a virus with long fibers. Notably, incorporation of the Ad5 fiber-derived domain in the targeted Ad43 vector did not compromise the virus' antigenic profile because the vector's infectivity was unaffected by Ad5-neutraling Abs. Therefore, the therapeutic derivatives of this vector prototype would face little risk of neutralization in patients with preexisting anti-Ad5 Abs.

In conclusion, this study showed that systemically delivered Ad43 demonstrates low levels of random transduction and relatively quick clearance from the host tissues. It also demonstrated the feasibility of manipulating the virus' specificity for primary receptor while retaining Ad43's unique antigenicity. Together with the previously reported low seroprevalence of Ad43 in humans, these findings identify this virus a promising candidate for future development of gene therapy vectors.

## MATERIALS AND METHODS

### Sequencing of the Ad43 genome, genome annotation, and global pairwise alignment

The stock of Ad43 was purchased from the American Type Culture Collection, ATCC (Manassas, VA, catalog #VR-1305). Viral DNA isolated from the virus amplified in 293 cells was sequenced and the reads were assembled using ContigExpress component of Vector NTI software (Life Technologies, Carlsbad, CA). The J. Craig Venter Institute Annotation Service analyzed the resultant contig using their adenovirus automated annotation algorithm based on homology predictions (http://gsc.jcvi.org/projects/gsc/adenovirus/index.php). A global pairwise sequence comparison of Ad43 with other human Ads was performed using the sequence alignment program mVISTA LAGAN (http://genome.lbl.gov/vista/mvista/submit.shtml). The GenBank accession numbers for analyzed viral genomes are shown in Supplementary Figure S1.

### Cells and reagents

Human embryonic kidney 293 and 293T cells, Chinese hamster ovary CHO cells, and breast carcinoma SK-BR-3 cells were obtained from the ATCC. 293A cells were obtained from Life Technologies. Human breast MDA-MB-231 and ovarian SKOV3.ip1 carcinomas were provided by Janet Price (MD Anderson Cancer Center, Houston), and CHO-C2 cells that express the C2 isoform of CD46 were provided by Andre Lieber (University of Washington, Seattle). 293/Her2 and MDA-MB-231/Her2 cells were developed in our laboratory [4, 36]. All cell lines were maintained as recommended by the vendor or as previously reported. The cells were authenticated at M. D. Anderson's Characterized Cell Line Core Facility using Short Tandem Repeat (STR) method. The cultures were tested for mycoplasma contamination monthly.

293F43 and 293F_43S5_11F_Her2:7_ cell lines constitutively expressing the wt Ad43 fiber or F_43S5_11F_Her2:7_ chimera were derived from 293 cells by transfection with pFXhAd43 and pFusion27_43S5_ plasmids, respectively, and selection on G418. The level of fiber expression in selected clones was quantitated using western blotting of cell lysates with anti-fiber mouse Ab 4D2 [50].

RGD-containing peptide (GRGDSP) was obtained from Sigma (St. Louis, MO). The Ad35 fiber knob protein was a gift from Andre Lieber. The Z_her2-7_ affibody and anti-Ad5 neutralizing Ab were produced as reported previously [4, 51]. The Ad43 knob protein was expressed in *Escherichia coli* using pET20b plasmid vector (EMD Millipore, Billerica, MA) and purified by chromatography using the protocols reported previously [52]. Recombinant soluble human CD46 protein comprising the extracellular component of the receptor was produced and purified as previously described [53].

### Genetic engineering

The cloning schemas, DNA maps, and sequences of the fiber- and fiber chimeras- expressing plasmids for the generation of 293F43 and 293F_43S5_11F_Her2:7_ cell lines and transient protein expression; the shuttle plasmids for the E1 region replacement; the fiber and fiber chimeras shuttle plasmids; and the rescue plasmids containing Ad genomes are available upon request.

### Viruses

The genomes of recombinant Ad43 and Ad35 vectors were assembled in rescue plasmids using homologous recombination in *Escherichia coli* strain BJ5183 between the corresponding shuttle and rescue plasmids, as previously described [36]. The genes of the E1 regions within these genomes were replaced with a CMV promoter–driven expression cassette containing the TL transgene, which encodes a genetic fusion of the herpes simplex virus thymidine kinase and firefly luciferase, and the E4 orf6 were replaced with the Ad5 E4 orf6. The Ad genomes were released from the corresponding rescue plasmids by restriction endonuclease digestion and used for transfection. The viruses containing wt fibers were rescued in 293 cells and then propagated in 293A cells.

The Ad43 vectors with chimeric fibers were initially rescued and propagated in 293F43 line 20. This yielded mosaic virions containing both the wt and chimeric fibers. To obtain vector preparation containing only the chimeric fibers, 293A cells were infected with these mosaic viruses. These infections were done at the same multiplicity of infection (MOI) of 3 infectious units (IU)/cell using infectious titers of mosaic Ad preparations determined in 293A cells by spot assay [54]. In brief, 293A cells seeded in 24-well plates were infected with serial dilutions of viral preparations, the infection was allowed to develop for 24 h, the individual cells in which productive infection has developed were identified by staining with anti-hexon Ab and counted.

The construction of Ad5TL-an E1-deleted Ad vector expressing a dual reporter gene TL-was previously described [36].

All Ads were purified by centrifugation in CsCl gradients, dialyzed against a buffer containing 10 mM Tris (pH 8.0), 50 mM NaCl, 2 mM MgCl, and 10% glycerol, and stored at -80°C. The particle titers of viral preparations were determined by measuring the total protein concentration, as previously described [36]: 1 mg of total Ad protein per milliliter was considered equivalent to 4 × 10^12^ VPs/ml.

### Surface plasmon resonance

Biosensor studies in which Ad virions were probed with human FX were performed essentially as described in our recent report [4].

### Studies in mice and experiments using murine tissues

All experiments involving animals were done according to protocols approved by the Institutional Animal Care and Use Committee of The University of Texas MD Anderson Cancer Center and followed National Institutes of Health and United States Department of Agriculture guidelines.

To study the *in vivo* patterns of transgene expression by Ad vectors, 6- to 8-week-old female NCr nu/nu mice (Taconic, Hudson, NY) were injected intravenously (tail vein) with either phosphate-buffered saline (PBS) or 4 × 10^10^ VPs in 100 μl of PBS. Forty-eight hours later, the mice were anesthetized by isoflurane inhalation and injected in the tail vein with a solution of D-luciferin (Caliper, Hopkinton, MA). The optical and X-ray images of mice were acquired using IVIS Lumina XR imaging system (PerkinElmer, Waltham, MA) and overlaid using Living Image software version 4.3 (PerkinElmer). The bioluminescence signals measured within the selected regions of interest were analyzed using Living Image software.

Twenty-four hours later, the mice were euthanized by CO_2_ inhalation, individual organs were collected, and homogenized. Aliquots of homogenized tissues were lysed in reporter lysis buffer (Promega, Madison, WI) and Fluc activity in supernatants was measured in a Sirius luminometer (Berthold, Pforzheim, Germany) using the luciferase assay system (Promega). The Wilcoxon rank-sum test was used to compare reporter activities by each vector for each tissue type using SAS 9.2 software (SAS Institute, Cary, NC).

To establish patterns of biodistribution of Ad particles, the mice were injected intravenously (tail vein) with 4 × 10^10^ VP of one of the tested Ads and killed at 1 h, 24 h, or 72 h after injection. After perfusion of animals with heparin-containing PBS, the tissue collection and lysis were performed. The 100-fold dilutions of tissue lysates in water were used as templates for quantitative PCR. Ad genomes were detected using primers and 6-carboxyfluorescein (6FAM)-labeled probes complementary to the E4 region and DNA polymerase gene, respectively. Reactions were run in an ABI 7500 instrument and the data were processed using ABI 7500 software (Life Technologies). Generalized linear models were used to predict and contrast the log10 copy numbers of viral DNA using tissue type, time point, and vector type. The Kruskal-Wallis test was used to compare Ad DNA copy numbers between time points separately by virus and tissue type. No adjustment for multiplicity was made.

### *In vitro* gene transfer

Transduction of cells was performed as previously described [4]. Briefly, cells were incubated for 10 minutes with either medium alone or medium containing affibody, recombinant knobs, sCD46, or lysates of cells expressing fibers. Lysates of the fiber-expressing cells were prepared by three freeze-thaw cycles and the fiber concentration in the lysates was determined by western blotting and normalized prior to incubation with the cells. Next, the cells were infected for 30 minutes with one of the Ad vectors. The medium was replaced with fresh medium and incubation was continued for 24 hours, after which the cells were lysed using reporter lysis buffer (Promega). Fluc activity in cell lysates was measured as described above.

In gene transfer experiments involving RGD-peptide and EDTA, the cells were incubated with RGD-peptide or treated with EDTA (2 mg/ml and 10 mM, respectively) in DMEM/F12 on ice for 1 h, and then Ad vectors diluted in 4% FSC-DMEM/F12 were added and cells were incubated on ice (to prevent virus internalization) for another hour. The unbound virus was removed by washing with the ice-cold medium and the cells were transferred to 37°C to allow virus internalization.

In the virus neutralization assay, the viruses were mixed with anti-Ad5 mouse serum that had been diluted in PBS prior to being added to cells.

### Flow cytometry

Cell attachment by the targeted fiber was detected according to the previously described protocol [36]. In brief, the cells were incubated with aliquots of cleared lysates of 293T cells either mock-transfected or transfected with the plasmid that expresses the fiber (wt or chimeric). The cell-bound fibers were detected with 4D2 mAbs and secondary goat anti-mouse IgG Ab labeled with Alexa Fluor 488 (Life Technologies). Cells were analyzed using a FACSCalibur instrument (BD Biosciences, Franklin Lakes, NJ).

### Western blotting and silver staining

The fiber proteins in cell lysates were analyzed by western blotting as previously reported [55]: the blotting membranes were probed with monoclonal anti-fiber Ab 4D2 [50] followed by labeled goat anti-mouse IgG Ab conjugated with near-infrared fluorescence dye IRDye800, the membranes were scanned in Odyssey scanner (Li-COR, Lincoln, NE) and images were processed using Image Studio 2.0 software. To determine the protein composition of the virions, samples containing 10^10^ purified VP were separated by sodium dodecyl sulfate polyacrylamide gel electrophoresis (SDS-PAGE) and the gels were stained using a PageSilver silver staining kit (Thermo Fisher Scientific, Waltham, MA) according to the manufacturer's recommendations.

### Statistical analysis

In the virus biodistribution studies, Wilcoxon rank-sum and Kruskal-Wallis tests were used to compare virus copy numbers by tissue type, virus, and/or time point. These analyses were performed using SAS 9.2 for Windows (SAS Institute Inc., Cary, NC). To predict the percentage of Ad43 virions incorporating various numbers of F_43S5_11F_Her2:7_ chimera, calculations were done using a binomial distribution model. The reporter expression data and western blotting data on the expression of the Ad43 fiber and fiber chimeras were processed using analysis of variance (ANOVA) and the Tukey procedure to adjust for multiple comparisons.

## SUPPLEMENTARY MATERIALS FIGURE AND TABLE




