# Supplementary material for: Native and engineered tropism of vectors derived from a rare species D adenovirus serotype 43
Source: Oncotarget. 2016 Jul 23;7(33):53414–29. doi: 10.18632/oncotarget.10800 (PMC5288196; doi:10.18632/oncotarget.10800)
Supplement: Supplementary file 1 [file oncotarget-07-53414-s001.pdf]

Native and engineered tropism of vectors derived from a rare species D adenovirus serotype 43

Supplementary Materials

Supplementary Table S1: Genes of Ad43. See Supplementary_Table_S1

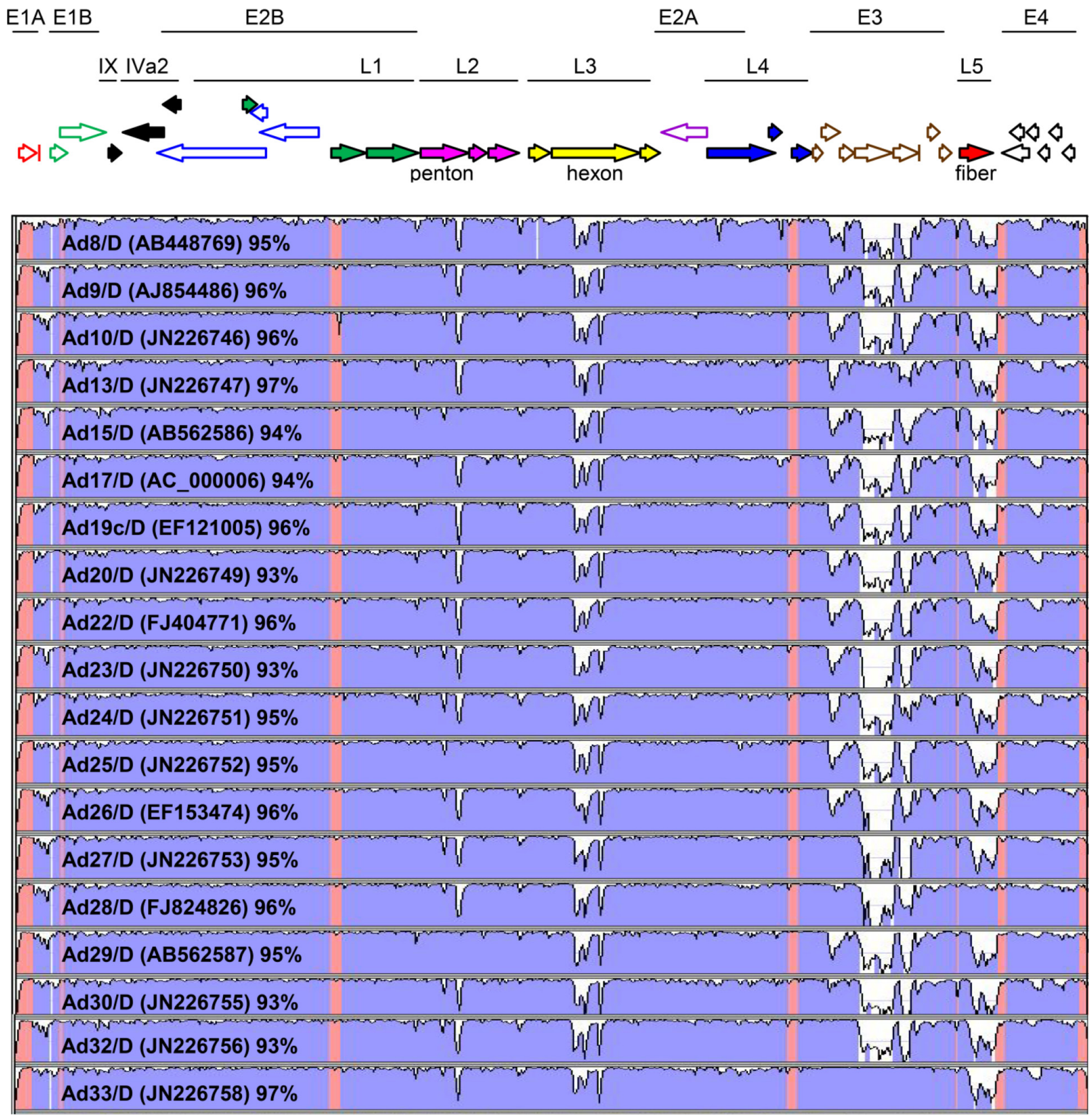
E1A
E1B
E2B
E2A
E3
E4
IX
IVa2
L1
L2
L3
L4
L5
penton
hexon
fiber
Ad8/D (AB448769) 95%
Ad9/D (AJ854486) 96%
Ad10/D (JN226746) 96%
Ad13/D (JN226747) 97%
Ad15/D (AB562586) 94%
Ad17/D (AC_000006) 94%
Ad19c/D (EF121005) 96%
Ad20/D (JN226749) 93%
Ad22/D (FJ404771) 96%
Ad23/D (JN226750) 93%
Ad24/D (JN226751) 95%
Ad25/D (JN226752) 95%
Ad26/D (EF153474) 96%
Ad27/D (JN226753) 95%
Ad28/D (FJ824826) 96%
Ad29/D (AB562587) 95%
Ad30/D (JN226755) 93%
Ad32/D (JN226756) 93%
Ad33/D (JN226758) 97%

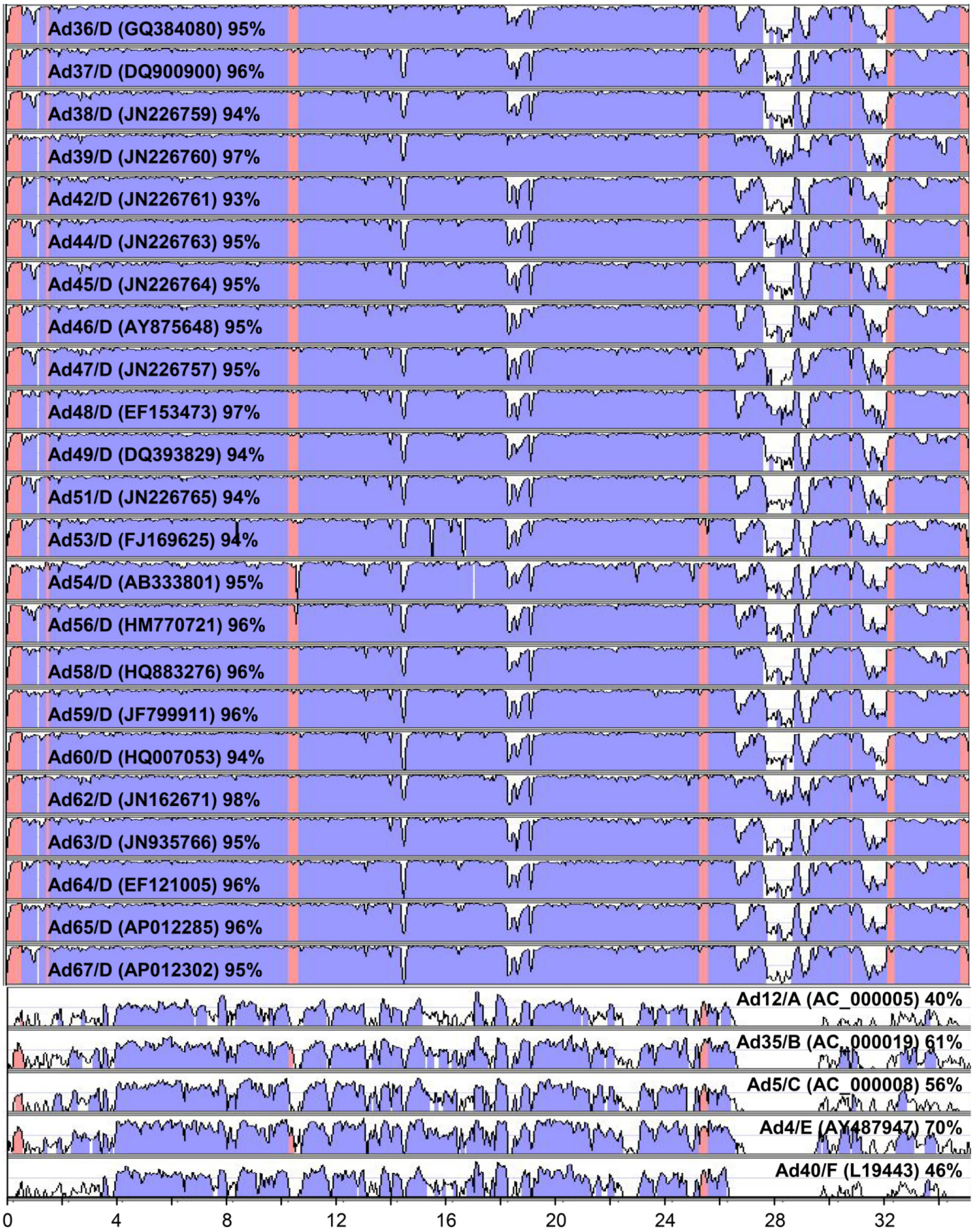

Supplementary Figure S1: Comparison of Ad43 genome with the genomes of other species D Ads and representatives of species A, B, C, E and F. The percent of sequence conservation at a given position (50% to 100% range) is shown by the height of the graph. The graphs show the coding and noncoding regions of the genomes in blue and pink, respectively. The genes within each region are shown in the same pattern/color. The major gene regions and genes of Ad43 are shown above the graphs. The numbers below the graphs show the genome coordinates in kilobase pairs. Each graph is identified by the Ad serotype, species, GenBank number, and percent of homology to Ad43. The Ad43 genome is 35,073 base pairs(bp)–long with the nucleotide composition of 22.5% A, 20.3% T, 28.6% C, and 28.6% G. The 57.2% GC content is in the 57%–59% range previously reported for other members of species D. The 5′ and 3′ ends are composed of 133 bp-long inverted terminal repeats (ITR). The Ad43 genome contains four early, two intermediate and five late transcription units, and includes 37 open reading frames (ORFs) and 5 hypothetical ORFs whose predicted products have no known analogues (Supplementary Table S1).
