# Supplementary material for: Native and engineered tropism of vectors derived from a rare species D adenovirus serotype 43
Source: Oncotarget. 2016 Jul 23;7(33):53414–29. doi: 10.18632/oncotarget.10800 (PMC5288196; doi:10.18632/oncotarget.10800)
Supplement: Supplementary file 2 [file oncotarget-07-53414-s002.docx]

**Table S1. Genes of Ad43.**

| **Region** | **Gene product** | **Location**^a^ | **Strand** | **Protein**  **length (aa)** | **Molecular**  **weight (kDa)**^b^ |
| --- | --- | --- | --- | --- | --- |
| E1A | E1A 21K protein | 562 - 925, 1206 - 1417 | + | 191 | 21.5 |
|  | E1A 28K protein | 562 - 1111, 1206 - 1417 | + | 253 | 28.5 |
| E1B | E1B 19K protein | 1570 - 2118 | + | 182 | 21.3 |
|  | E1B 55K protein | 1875 - 3362 | + | 495 | 55.0 |
| Intermediate | pIX protein | 3447 - 3854 | + | 135 | 14.0 |
|  | encapsidation protein IVa2 | 3897 - 5230, 5509 - 5521 | - | 448 | 50.8 |
|  | IVa2-2 hypothetical protein | 5194 - 5760 | - | 188 | 20.0 |
| E2B | DNA polymerase | 5000 - 8521, 13479 - 13487 | - | 1176 | 134.0 |
|  | terminal protein precursor pTP | 8320 - 10221, 13479 - 13487 | - | 636 | 73.2 |
|  | hypothetical protein | 8025 - 8558 | - | 178 | 19.2 |
| L1 | hypothetical protein | 6144 - 6413 | + | 90 | 9.7 |
|  | hypothetical protein | 7781 - 8248 | + | 155 | 16.8 |
|  | 52/55K protein | 10637 - 11758 | + | 373 | 42.3 |
|  | pIIIa penton-associated protein | 11781 - 13460 | + | 559 | 62.2 |
| L2 | penton base | 13514 - 15076 | + | 520 | 58.9 |
|  | core protein precursor pVII | 15080 - 15670 | + | 196 | 21.9 |
|  | core protein pV | 15703 - 16701 | + | 332 | 38.0 |
|  | core protein precursor pX | 16731 - 16955 | + | 74 | 8.3 |
| L3 | capsid protein precursor pVI | 17011 - 17715 | + | 234 | 25.6 |
|  | hexon protein | 17756 - 20611 | + | 951 | 107.4 |
|  | protease | 20614 - 21243 | + | 209 | 23.7 |
| E2A | DNA binding protein | 21287 - 22756 | - | 489 | 55.0 |
|  | hypothetical protein | 23915 - 24160 | - | 81 | 9.4 |
| L4 | hexon assembly 100K protein | 22773 - 24971 | + | 732 | 82.4 |
|  | 22K protein | 24754 - 25167 | + | 137 | 16.0 |
|  | capsid protein precursor pVIII | 25496 - 26179 | + | 227 | 24.7 |
| E3 | 12.2K-like protein | 26180 - 26500 | + | 106 | 12.3 |
|  | CR1-alpha protein | 26454 - 27047 | + | 197 | 22.2 |
|  | gp19K-like protein | 27044 - 27514 | + | 156 | 17.7 |
|  | CR1-beta protein | 27537 - 28760 | + | 407 | 45.9 |
|  | CR1-gamma1 protein | 28787 - 29599 | + | 270 | 29.7 |
|  | RID-alpha protein | 29606 - 29881 | + | 91 | 10.6 |
|  | RID-beta protein | 29884 - 30270 | + | 128 | 14.7 |
|  | 14.7K-like protein | 30263 - 30652 | + | 129 | 14.7 |
| U | U protein | 30755 - 30904 | - | 49 | 6.1 |
| L5 | fiber protein (pIV) | 30920 - 31999 | + | 359 | 39.4 |
| E4 | Orf 6 34K protein | 32276 - 33154 | - | 292 | 34.2 |
|  | 17K protein | 32532 - 32987 | - | 151 | 17.2 |
|  | Orf 4 | 33084 - 33446 | - | 120 | 14.2 |
|  | Orf 3 | 33450 - 33803 | - | 117 | 13.7 |
|  | Orf 2 | 33800 - 34192 | - | 130 | 14.6 |
|  | Orf 1 | 34233 - 34610 | - | 125 | 14.1 |

^a^ Genes within Ad43 genome were identified using the J. Craig Venter Institute Annotation Service (<http://gsc.jcvi.org/projects/gsc/adenovirus/index.php>.

^b^ Molecular weights of Ad43 proteins were predicted on the basis of their amino acid sequences.
